# Neutral Sphingomyelinase 2-Dependent Secretory Pathway May Contribute to Extracellular Hepatitis A Virus RNA Levels

**DOI:** 10.3390/v18101108

**Published:** 2026-10-08

**Authors:** Reina Sasaki-Tanaka, Tatsuo Kanda, Hanako Yamazaki, Shun Yamazaki, Ryo Jimbo, Tomoaki Yoshida, Naruhiro Kimura, Hiroyuki Abe, Takeshi Yokoo, Akira Sakamaki, Hiroteru Kamimura, Kenya Kamimura, Hiroaki Okamoto, Shuji Terai

**Affiliations:** 1 Division of Gastroenterology and Hepatology, Graduate School of Medicine, Dentistry and Health Sciences, Niigata University, 1-757 Asahimachi-dori, Chuo-ku, Niigata 951-8510, Japan; kandatatsuo@gmail.com (T.K.); hanakoy87@outlook.jp (H.Y.); arsenal0517@yahoo.co.jp (S.Y.); ryo.toda.0529@gmail.com (R.J.); tomoakiy@med.niigata-u.ac.jp (T.Y.); nkimura@med.niigata-u.ac.jp (N.K.); hiroyukiabe@med.niigata-u.ac.jp (H.A.); t-yokoo@med.niigata-u.ac.jp (T.Y.); saka-a@med.niigata-u.ac.jp (A.S.); kenya-k@med.niigata-u.ac.jp (K.K.); terais@med.niigata-u.ac.jp (S.T.); 2 Division of Gastroenterology and Hepatology, Uonuma Institute of Community Medicine, Niigata University Medical and Dental Hospital, Minamiuonuma 949-7302, Japan; 3 Department of General Medicine, School of Medicine, Niigata University, Niigata 951-8520, Japan; 4 Division of Virology, Department of Infection and Immunity, Graduate School of Medicine, Jichi Medical University, Shimotsuke 329-0498, Japan; hokamoto@jichi.ac.jp

**Keywords:** hepatitis A, nSMase2, ESCRT-independent pathway, GW4869, SMPD3

## Abstract

Hepatitis A virus (HAV) infection is a major cause of acute viral hepatitis worldwide. However, no effective antiviral therapy is currently available. HAV manipulates host secretory pathways, including the endosomal sorting complex required for transport (ESCRT)-dependent pathways, to facilitate viral propagation. Nevertheless, whether HAV also utilizes alternative secretory pathways remains unclear. Here, we provide evidence suggesting that an ESCRT-independent pathway may contribute to extracellular HAV RNA levels in Huh7 cells infected with HAV genotype IB (HM175-18f) and genotype IIIA (HA11-1299). Treatment with the ESCRT-independent pathway inhibitor GW4869 significantly reduced extracellular HAV RNA levels. GW4869 did not significantly affect HAV subgenomic RNA replication. These results suggest that the neutral sphingomyelinase 2 (nSMase2)-dependent ESCRT-independent pathway may contribute to extracellular HAV RNA levels rather than intracellular HAV RNA replication. Consistent with these findings, knockdown of sphingomyelin phosphodiesterase 3, which encodes SMase2, also significantly reduced extracellular HAV RNA levels. These findings suggest that an nSMase2-dependent ESCRT-independent extracellular vesicle pathway may contribute to extracellular HAV RNA levels.

## 1. Introduction

Hepatitis A virus (HAV) is a single-stranded, positive-sense RNA virus [1]. HAV is among the major causes of acute viral hepatitis [2]. It is estimated that 159 million cases of HAV infection occurred globally in 2019, resulting in approximately 39,000 deaths [2]. HAV genotype III infection is frequently associated with cholestasis and prolonged hepatitis and is associated with more severe clinical manifestations than HAV genotype I infection [3], although the underlying mechanisms remain unclear. Given that there is currently no universal vaccination program against HAV infection in Japan and that the number of people without HAV immunity is increasing because of improvements in hygienic environments [4], the risk of HAV outbreaks may increase in the future. Therefore, a better understanding of the molecular mechanisms of HAV infection is needed to develop new effective antiviral therapies.

Extracellular vesicles (EVs) are cell-derived, membrane-enclosed particles that serve as key mediators of intercellular communication through the transfer of diverse bioactive cargos, including nucleic acids, proteins, and lipids. Although EVs can be classified by their biogenesis, the MISEV2023 guidelines recommend using size-based terminology, such as small EVs (sEVs, <200 nm) and medium/large EVs (m/lEVs, ≥200 nm), when biogenesis cannot be reliably determined [5]. EV terminology should be used cautiously because of the limitations of classification based on biogenesis and size.

Modulation of the molecular cargo packaged into EVs during viral infection determines whether these vesicles promote host antiviral defenses or support viral propagation. For instance, EVs derived from infected cells containing stimulators of interferon genes (STING) and viral mRNAs, whose main role may be to silence viral genes, can activate innate antiviral signaling in neighboring uninfected cells, thus limiting viral dissemination [6]. In contrast, several viruses commonly hijack and modify EV signaling pathways to increase the efficiency of viral spread to uninfected cells [7].

**Figure 1:**
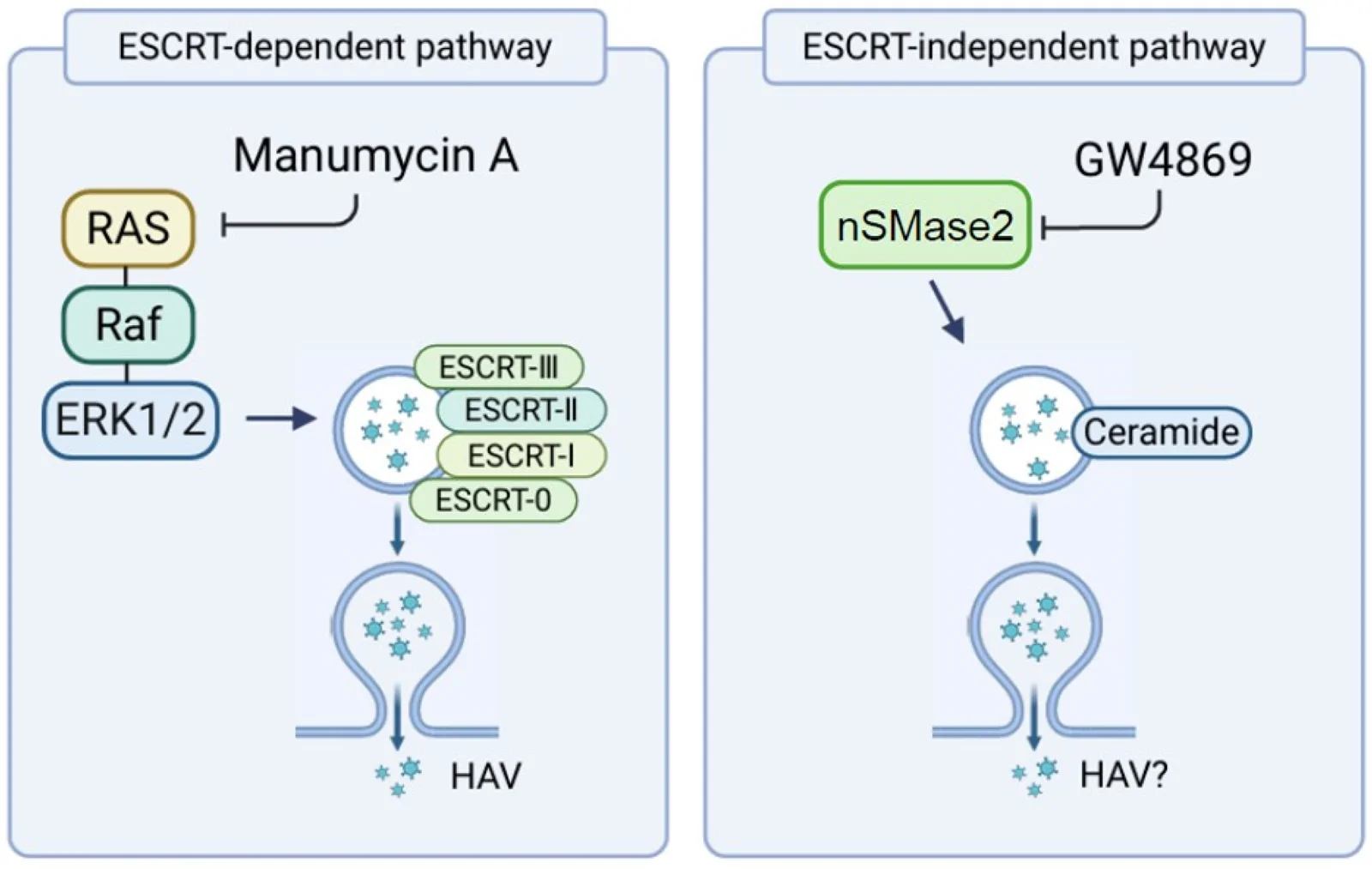
Schematic representation of the inhibitory targets of manumycin A and GW4869. Created in Biorender. Hiroyuki Abe. (2026) (https://app.biorender.com/illustrations/6996578a3d4e432df18fd3d3). ESCRT: endosomal sorting complex required for transport; RAS: rat sarcoma virus oncogene; Raf: rapidly accelerated fibrosarcoma; ERK1/2: extracellular signal-regulated kinase 1/2; nSMase2: neutral sphingomyelinase 2; HAV: hepatitis A virus.

EV biogenesis can proceed via the endosomal sorting complex required for transport (ESCRT)-dependent mechanisms involving the tumor susceptibility gene 101 (TSG101), apoptosis-linked gene 2-interacting protein X (ALIX), and charged multivesicular body protein 4 (CHMP4), as well as ESCRT-independent pathways mediated by ceramide synthesized by neutral sphingomyelinases (nSMases) [8], which are encoded by sphingomyelin phosphodiesterase 3 (SMPD3) [9]. Several pharmacological agents have been used to investigate distinct pathways involved in EV secretion. Obatoclax, a pan-Bcl-2 family inhibitor, has been reported to enhance EV secretion, at least in part, by increasing the generation of autophagosomes and amphisomes, which form through the fusion of accumulated autophagosomes with multivesicular endosomes [10]. In contrast, manumycin A inhibits rat sarcoma viral oncogene (Ras) farnesyltransferase [11] and has been reported to interfere with ESCRT-dependent pathways (Figure 1), whereas GW4869 inhibits nSMase2 [12] and has been reported to interfere with the nSMase2/ceramide-associated, ESCRT-independent pathway (Figure 1).

Because extracellular HAV RNA levels are considered largely ESCRT-dependent, we investigated whether an alternative nSMase2-dependent ESCRT-independent pathway also contributes to extracellular HAV RNA levels.

## 2. Materials and Methods

### 2.1. Cell Lines and Reagents

The human hepatoma cell line Huh7 and its derivative HuhT7 cells were used. Huh7 and HuhT7 cells were kindly provided by Prof. Ralf Bartenschlager and Prof. Verena Gauss-Müller, respectively [13,14]. HuhT7-HAV/Luc cells, which stably express HAV genotype IB (HM175-18f) subgenomic replicon RNA harboring the firefly luciferase (Fluc) gene, have previously been reported [15,16]. The African green monkey kidney cell line COS7 (JCRB 9127) was purchased from the Health Science Research Resources Bank (Osaka, Japan). COS7-HAV-IRES cells, which stably express the HAV internal ribosomal entry site (IRES), followed by the Fluc of the simian virus 40 (SV40) promoter plasmid pSV40-HAV-IRES, were used [17,18]. These cells were maintained in Roswell Park Memorial Institute medium (RPMI; Sigma-Aldrich, St. Louis, MO, USA) supplemented with 10% heat-inactivated fetal bovine serum (FBS; Sigma-Aldrich), 100 units/mL penicillin, and 100 μg/mL streptomycin (Sigma-Aldrich) in a 5% CO2 atmosphere at 37 °C.

The HAV genotype IIIA (HA11-1299) strain was described previously [11]. The HAV genotype IB (HM175-18f) strain, which was kindly provided by Prof. Stanley M. Lemon, was used for HAV infection in the present study [16].

Obatoclax (GX15-070), a pan-Bcl-2 family inhibitor, and GW4869, an inhibitor of nSMase2, were purchased from Selleck Biotech (Houston, TX, USA; Cat. Nos. S6709 and S7609, respectively). Manumycin A, a Ras farnesyltransferase inhibitor, was purchased from AdipoGen Life Sciences, Inc. (San Diego, CA, USA; Cat. No. AG-CN2-2000-M001).

Small interfering RNAs (siRNAs) against SMPD3 (si-SMPD3) and control siRNA (si-Control) were purchased from Thermo Fisher Scientific (Waltham, MA, USA; Cat. No. s30925) and Santa Cruz Biotechnology (Dallas, TX, USA; Cat. No. sc-37007), respectively.

### 2.2. Infection of Huh7 Cells with HAV Genotype IB (HM175-18f) and HAV Genotype IIIA (HA11-1299)

**Figure 2:**
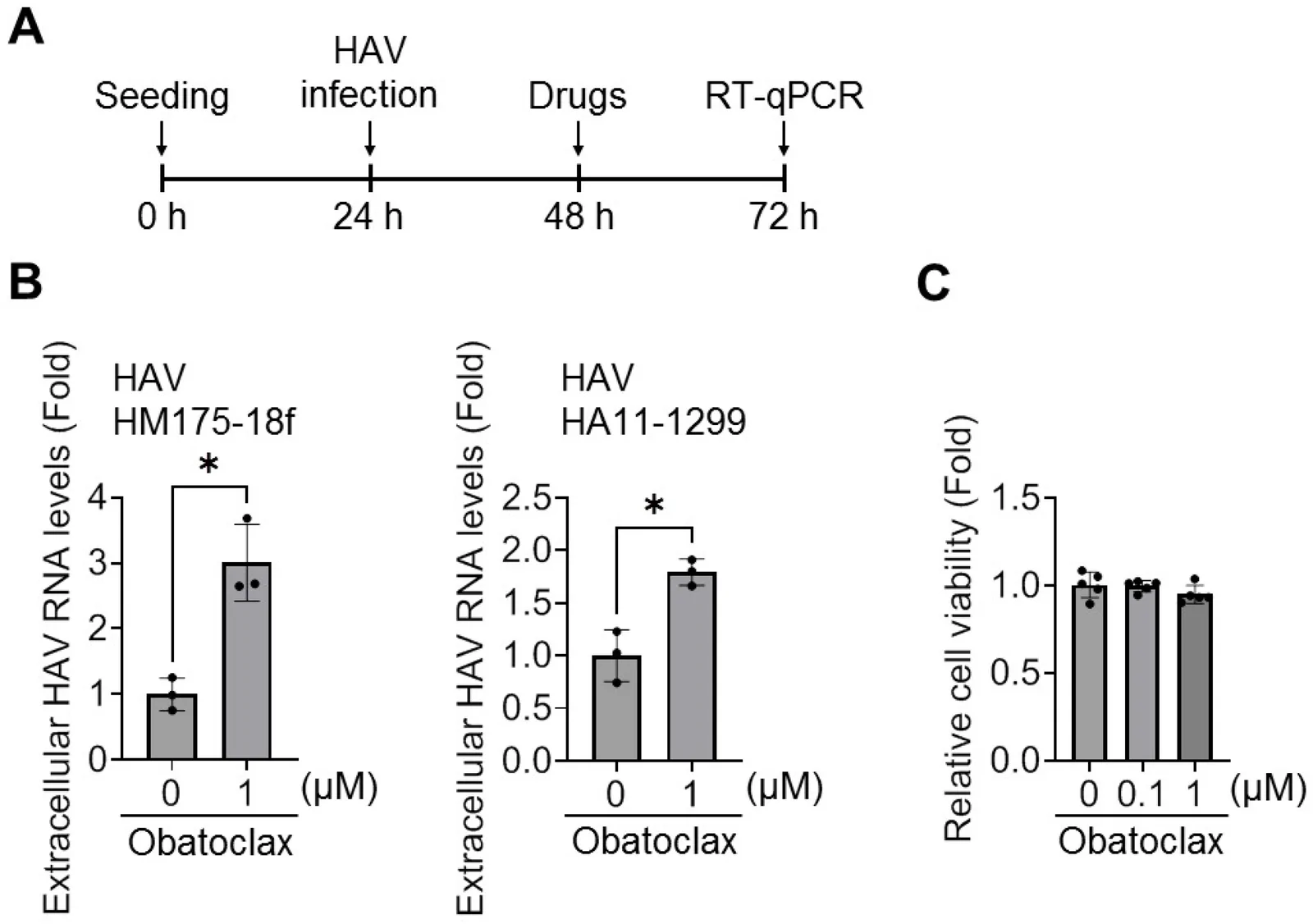
Obatoclax enhances extracellular hepatitis A virus (HAV) RNA levels. (**A**) Schematic diagram of the experimental timeline. (**B**) Effects of obatoclax on extracellular HAV RNA levels. Huh7 cells infected with HAV genotype IB (HM175-18f) and HAV genotype IIIA (HA11-1299) were treated with obatoclax for 24 h. Extracellular HAV RNA levels were measured by real-time RT-PCR. The data are presented as the means and standard deviations of three independent biological replicates obtained from at least three independent experiments. (**C**) Effects of obatoclax on Huh7 cell viability. Cells were treated with obatoclax at the indicated concentrations. Cell viability was measured via dimethylthiazol carboxymethoxyphenyl sulfophenyl tetrazolium (MTS) assays. Statistical significance was determined using Welch’s *t*-test: * *p* < 0.05.

**Figure 3:**
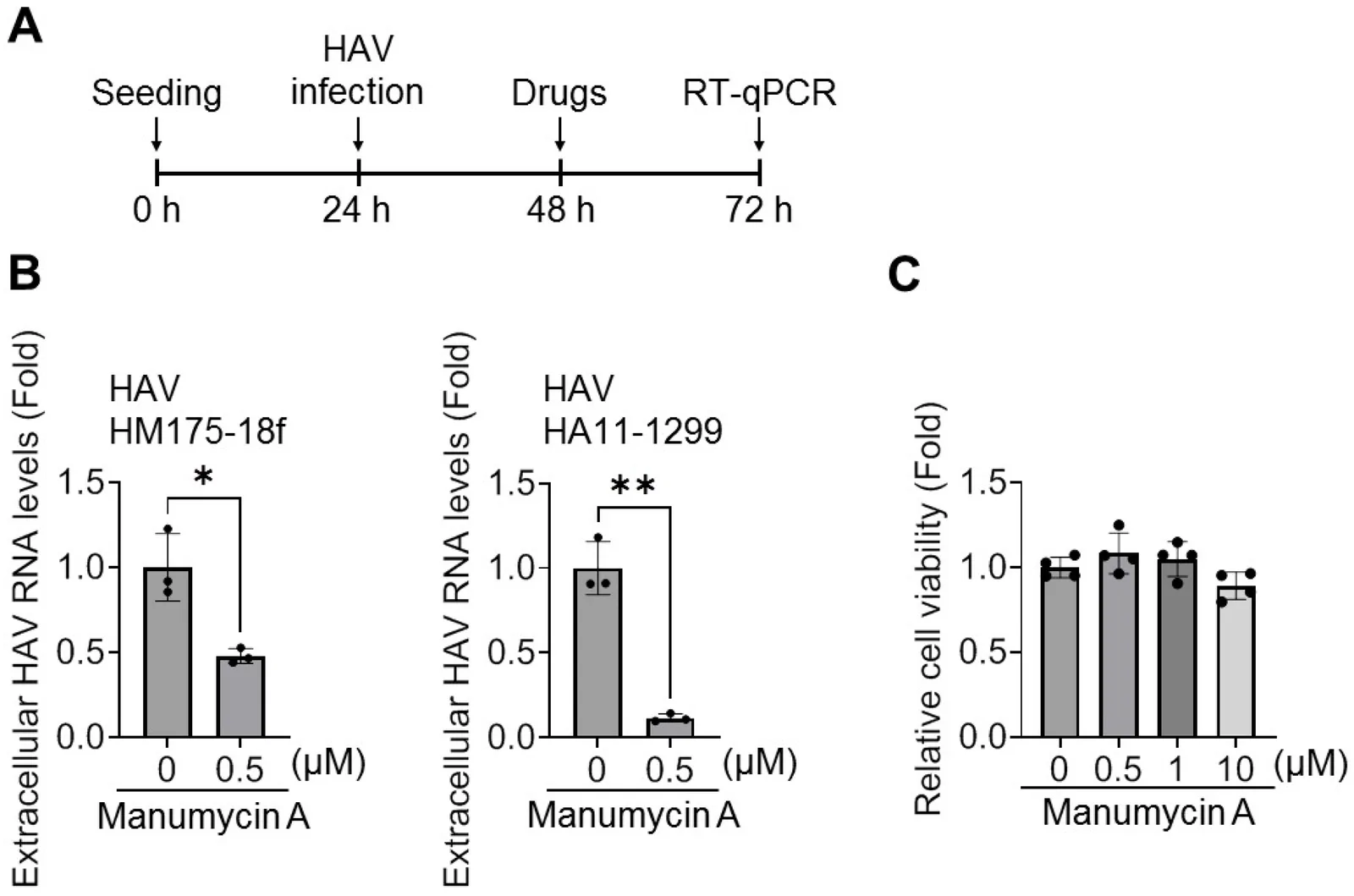
Manumycin A decreases extracellular hepatitis A virus (HAV) RNA levels. (**A**) Schematic diagram of the experimental timeline. (**B**) Effects of manumycin A on extracellular HAV RNA levels. Huh7 cells infected with HAV genotype IB (HM175-18f) and HAV genotype IIIA (HA11-1299) were treated with manumycin A for 24 h. Extracellular HAV RNA levels were measured by real-time RT-PCR. The data are presented as the means and standard deviations of three independent biological replicates obtained from at least three independent experiments. (**C**) Effects of manumycin A on Huh7 cell viability. Cells were treated with manumycin A at the indicated concentrations. Cell viability was measured via dimethylthiazol carboxymethoxyphenyl sulfophenyl tetrazolium (MTS) assays. Statistical significance was determined using Welch’s *t*-test: * *p* < 0.05, ** *p* < 0.01.

**Figure 4:**
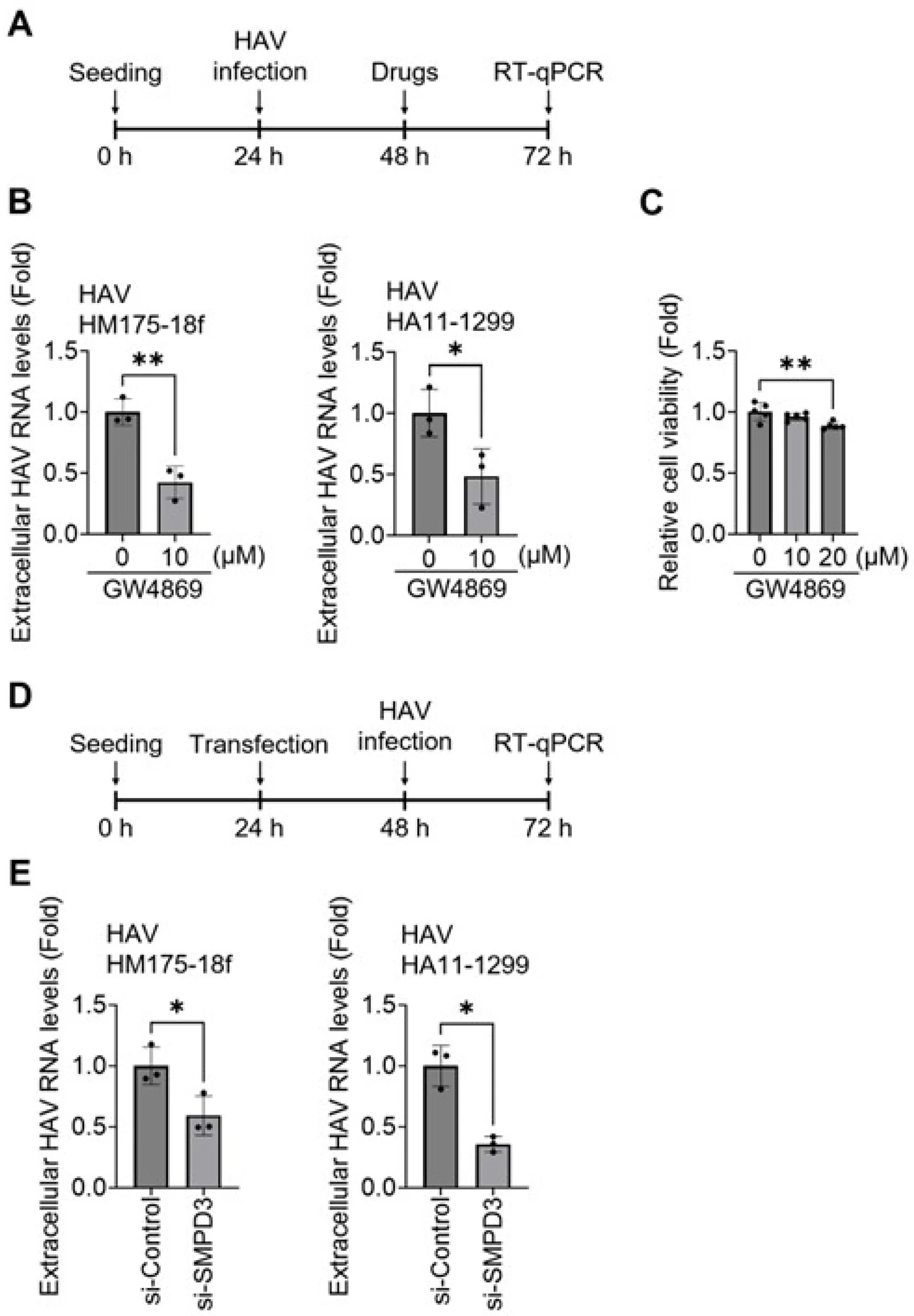
GW4869 decreases extracellular hepatitis A virus (HAV) RNA levels. (**A**) Schematic diagram of the experimental timeline. (**B**) Effects of GW4869 on HAV RNA levels in culture supernatant. Huh7 cells infected with HAV genotype IB (HM175-18f) or HAV genotype IIIA (HA11-1299) were treated with GW4869 for 24 h. Extracellular HAV RNA levels were measured by real-time RT-PCR. (**C**) Effects of GW4869 on Huh7 cell viability. Cells were treated with GW4869 at the indicated concentrations. Cell viability was measured via dimethylthiazol carboxymethoxyphenyl sulfophenyl tetrazolium (MTS) assays. (**D**) Schematic diagram of the experimental timeline. (**E**) Effects of knockdown of sphingomyelin phosphodiesterase 3 (SMPD3) on HAV RNA levels in culture supernatant. Huh7 cells were transfected with si-SMPD3 and si-Control, followed by infection with HAV genotype IB (HM175-18f) and HAV genotype IIIA (HA11-1299) for 72 h. Extracellular HAV RNA levels were measured by real-time RT-PCR. The data are presented as the means and standard deviations of three independent biological replicates obtained from at least three independent experiments. Statistical significance was determined using Welch’s *t*-test: * *p* < 0.05, ** *p* < 0.01.

Twenty-four hours before infection, Huh7 cells were seeded at a density of 6 × 10^5^ cells/well into 6-well plates (AGC TECHNO GLASS, Haibara, Shizuoka, Japan). The cells were subsequently washed twice with phosphate-buffered saline (PBS) (FUJIFILM Wako Pure Chemical Corporation, Tokyo, Japan) and infected with HAV genotype IB (HM175-18f) and HAV genotype IIIA (HA11-1299) at a multiplicity of infection (MOI) of 0.1 in serum-free medium [19]. The control cells remained uninfected by HAV. The HAV inoculum was incubated with hepatocytes for 24 h. The cells were subsequently washed once with PBS, after which 1 mL of RPMI containing 5% FBS was added. Afterward, 0 and 1 μM obatoclax, 0 and 0.5 μM manumycin A and 0 and 10 μM GW4869 were added to these cells. Obatoclax, manumycin A, and GW4869 were dissolved in dimethyl sulfoxide (DMSO) (Sigma-Aldrich, Burlington, MA, USA). The final concentrations of DMSO in the culture medium were 0.015754%, 0.0009081%, and 0.08658% for obatoclax, manumycin A, and GW4869, respectively. The 0 μM control groups received the same concentration of vehicles as the corresponding treatment groups. After 48 h of infection, HAV RNA levels in the culture supernatant were measured by real-time RT-PCR (Figure 2A, Figure 3A and Figure 4A).

### 2.3. Transfection of Small Interfering RNAs (siRNAs) into Huh7 Cells

The siRNAs, si-SMPD3 or si-Control were transfected into Huh7 cells using Effectene Transfection Reagent (Qiagen, Venlo, The Netherlands). Huh7 cells were plated at a density of approximately 6 × 10^5^ cells/well in 6-well plates and transfected with 50 nM si-SMPD3 or si-Control. After 24 h, the cells were infected with HAV genotype IB (HM175-18f) and HAV genotype IIIA (HA11-1299) at an MOI of 0.1 in serum-free RPMI. After 72 h of infection, extracellular HAV RNA in the culture supernatant in HAV-infected cells were quantified by real-time RT-PCR (Figure 4D).

### 2.4. Quantification of HAV RNA

For culture supernatants collected from HAV-infected or siRNA-SMPD3-treated cells, HAV RNA was quantified using the Easy Direct RT-qPCR Kit (TaKaRa Bio, Ohtsu, Shiga, Japan) and Hepatitis A Virus Primer/Probe Mix (TaKaRa Bio). TaqMan real-time PCR was performed under the following conditions: 90 °C for 3 min and 60 °C for 5 min, followed by 45 cycles of 95 °C for 5 s and 60 °C for 30 s on a StepOnePlus Real-Time PCR System (Applied Biosystems, Urayasu, Chiba, Japan). HAV RNA levels were determined by reference to a standard curve generated using HAV positive control RNA (TaKaRa Bio).

### 2.5. Cell Viability Assays

To evaluate cell viability, dimethylthiazol carboxymethoxyphenyl sulfophenyl tetrazolium (MTS) assays were performed using the CellTiter 96 Aqueous One-Solution cell proliferation assay (Promega, Madison, WI, USA). Enzyme activity was measured using a Bio-Rad iMark microplate reader (Bio-Rad, Hercules, CA, USA) at a wavelength of 490 nm, as previously described [20].

### 2.6. Assessment of Drug Effects on HAV Replication and Translation

**Figure 5:**
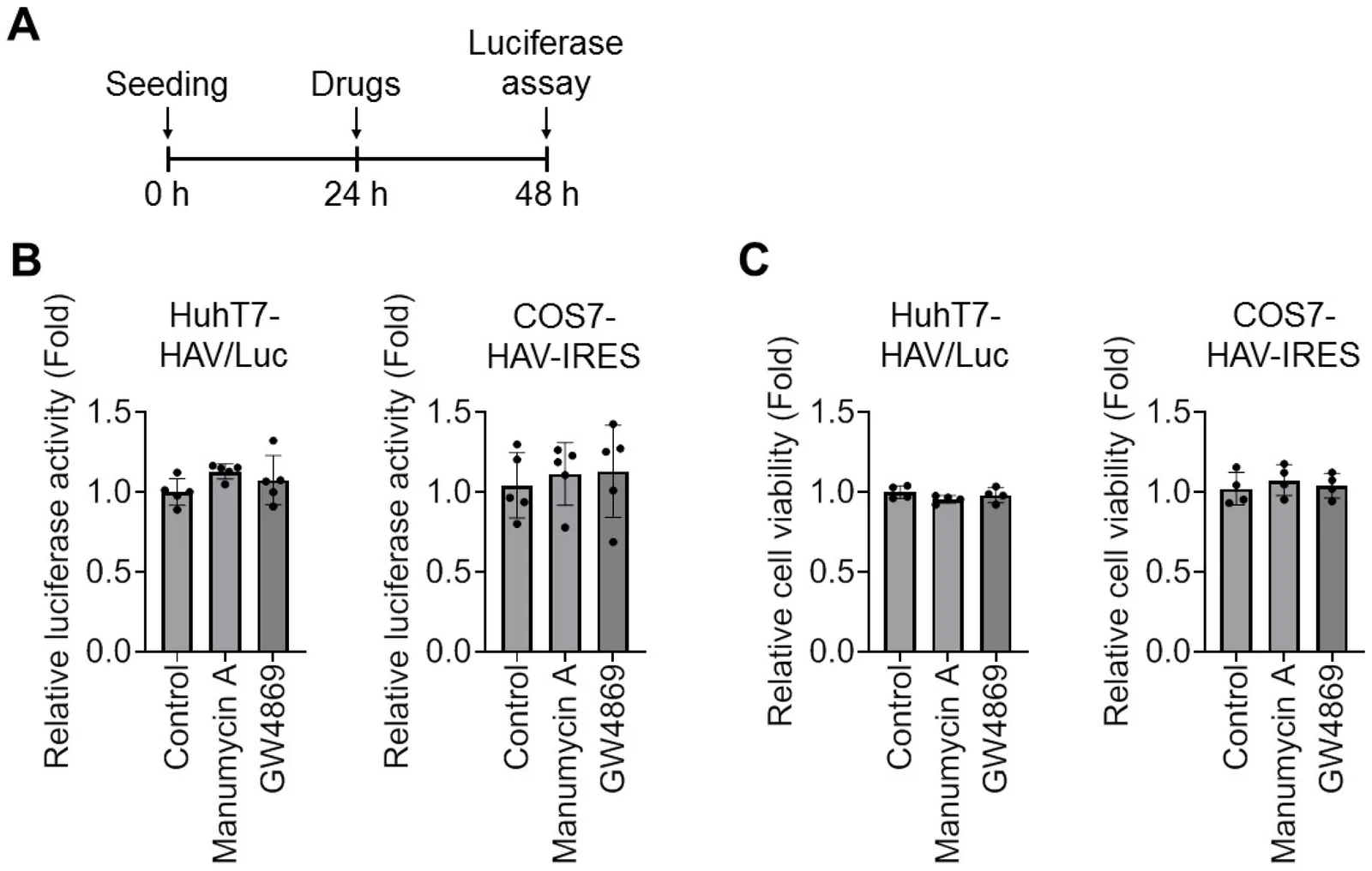
Manumycin A and GW4869 do not inhibit hepatitis A virus (HAV) HM175-18f genotype IB subgenomic replicon replication or internal ribosomal entry site (IRES)-mediated translation. (**A**) Schematic diagram of the experimental timeline. (**B**) Effects of manumycin A and GW4869 on HAV subgenomic replicon replication and HAV IRES-mediated translation. HuhT7-HAV/Luc cells and COS7-HAV-IRES cells were treated with 0.5 μM manumycin A and 10 μM GW4869. After 24 h of incubation, luciferase activity was determined. (**C**) The cytotoxicity of 0.5 μM manumycin A and 10 μM GW4869 on HuhT7-HAV/Luc cells and COS7-HAV-IRES cells. Cell viability was measured via dimethylthiazol carboxymethoxyphenyl sulfophenyl tetrazolium (MTS) assays. The data are expressed as the means and standard deviations of three independent biological replicates obtained from three independent experiments. Statistical significance was determined using one-way analysis of variance (ANOVA) followed by Tukey’s multiple-comparison test.

Briefly, HuhT7-HAV/Luc and COS7-HAV-IRES cells were plated at a density of approximately 6 × 10^5^ cells/well into 6-well plates. After 24 h of incubation, the HuhT7-HAV/Luc and COS7-HAV-IRES cells were washed twice with PBS, after which 1 mL of RPMI supplemented with 10% FBS was added. Afterward, 0 and 0.5 μM manumycin A and 0 and 10 μM GW4869 were added to the cells. The 0 μM control groups received the same concentration of vehicles as the corresponding treatment groups. After 24 h of incubation, the cells were harvested using reporter lysis buffer (Toyo Ink, Tokyo, Japan), and luciferase activity was determined using a luminometer (AB-2200-R, ATTO, Tokyo, Japan), as previously described (Figure 5A) [16].

### 2.7. Statistical Analysis

The data are expressed as the means ± standard deviations (SDs). Statistical analysis was performed using Welch’s *t*-test for comparisons between two groups and one-way analysis of variance (ANOVA) followed by Tukey’s multiple-comparison test for comparisons among three groups. Individual measurement values are shown as individual dots in the figures. *p*  <  0.05 was considered to indicate statistical significance.

## 3. Results

### 3.1. The EV Activator Obatoclax Significantly Upregulated HAV Genotype IB (HM175-18f) and HAV Genotype IIIA (HA11-1299) RNA Levels in Culture Supernatant from HAV-Infected Huh7 Cells

We first examined the effects of obatoclax, a pan-Bcl-2 family inhibitor [10], on HAV RNA levels in culture supernatant from HAV genotype IB (HM175-18f)- and HAV genotype IIIA (HA11-1299)-infected Huh7 cells after 48 h of infection (Figure 2A,B). HAV RNA levels were significantly upregulated by obatoclax in culture supernatant from Huh7 cells infected with both HAV genotype IB (HM175-18f) and HAV genotype IIIA (HA11-1299). At these concentrations, obatoclax did not induce cytotoxicity in Huh7 cells after 48 h (Figure 2C).

### 3.2. ESCRT-Dependent Pathway Inhibitor Manumycin A Significantly Decreases Extracellular HAV RNA Levels in Culture Supernatant from HAV-Infected Huh7 Cells

We examined the effects of manumycin A, which has been reported to interfere with ESCRT-dependent pathways [11], on HAV RNA levels in culture supernatant from Huh7 cells infected with HAV genotype IB (HM175-18f) and HAV genotype IIIA (HA11-1299) after 48 h of infection (Figure 1). After 48 h of infection, the HAV RNA levels in these cells were measured using real-time RT-PCR (Figure 3A,B). Extracellular HAV RNA levels were significantly inhibited by manumycin A in culture supernatant from both HAV genotype IB (HM175-18f)- and HAV genotype IIIA (HA11-1299)-infected Huh7 cells. At these concentrations, manumycin A did not induce cytotoxicity in Huh7 cells after 48 h (Figure 3C).

### 3.3. Pharmacological Inhibition of nSMase2/Ceramide-Associated Pathways Decreases Extracellular HAV RNA Levels in Culture Supernatant from HAV-Infected Huh7 Cells

Next, we examined the effects of GW4869, which has been reported to interfere with nSMase2/ceramide-associated pathways [12], on the RNA levels of HAV genotype IB (HM175-18f) and HAV genotype IIIA (HA11-1299) in HAV-infected Huh7 cells for 48 h of infection (Figure 1). After 48 h of infection, extracellular HAV RNA levels were significantly inhibited by GW4869 in culture supernatant from both HAV genotype IB (HM175-18f)- and HAV genotype IIIA (HA11-1299)-infected Huh7 cells (Figure 4A,B). At these concentrations, GW4869 did not induce cytotoxicity in Huh7 cells after 48 h (Figure 4C).

To further confirm the involvement of the ESCRT-independent pathway, we performed siRNA-mediated knockdown of SMPD3 and evaluated its effects on extracellular HAV RNA levels in culture supernatant from Huh7 cells infected with HAV genotype IB (HM175-18f) and HAV genotype IIIA (HA11-1299) (Figure 4D,E). Extracellular HAV RNA levels were significantly inhibited by si-SMPD3 in culture supernatant from both HAV genotype IB (HM175-18f)- and HAV genotype IIIA (HA11-1299)-infected Huh7 cells (Figure 4E).

### 3.4. Manumycin A and GW4869 Have No Significant Effects on HAV Translation or Replication

We examined the effects of manumycin A and GW4869 on HAV replication in HuhT7-HAV/Luc cells. After 24 h of incubation, manumycin A and GW4869 treatment did not significantly affect the replication of the HAV genotype IB (HM175-18f) subgenomic replicon (Figure 5A,B). At these concentrations, neither manumycin A nor GW4869 induced cytotoxicity in HuhT7-HAV/Luc cells during the 24 h treatment period (Figure 5C).

Similarly, we examined the effects of manumycin A and GW4869 on HAV IRES-dependent translation activity in COS7-HAV-IRES cells after 24 h of treatment. Neither manumycin A nor GW4869 significantly affected the IRES-dependent translation activity of HAV genotype IB (HM175-18f) (Figure 5A,B), and no cytotoxicity was observed in COS7-HAV-IRES cells during the 24 h treatment period (Figure 5C).

## 4. Discussion

In the present study, we investigated the potential contribution of an nSMase2-dependent ESCRT-independent pathway to extracellular HAV RNA levels. Pharmacological inhibition of the nSMase2/ceramide-associated pathway using GW4869 significantly reduced extracellular HAV RNA levels in both HAV genotype IIIA (HA11-1299) and HAV genotype IB (HM175-18f). GW4869 did not significantly affect subgenomic RNA replicon or IRES-dependent translation of HAV genotype IB (HM175-18f). These findings suggest that this pathway may contribute to extracellular HAV RNA levels.

EV biogenesis and release are regulated by both ESCRT-dependent and ESCRT-independent pathways [21]. Previous reports have suggested that the ESCRT-dependent machinery is involved in the release of several enveloped viruses, including human immunodeficiency virus (HIV), hepatitis C virus (HCV), Ebola virus, and classical swine fever virus (CSFV) [22,23,24,25]. Furthermore, cytomegalovirus (HCMV) and enterovirus 71 (EV71) have been reported to interact with both ESCRT-dependent and ESCRT-independent pathways during virus release [26,27].

Although HAV is traditionally considered a non-enveloped virus, quasi-enveloped HAV particles enclosed within EV-like membranes have been detected in circulation, suggesting that HAV may be associated with pathways involved in EV biology [28]. Components of the ESCRT machinery, including ESCRT-0, ESCRT-I, and ESCRT-III, have been implicated in HAV particle release [29,30]. Furthermore, HAV VP1pX–ALIX interactions have been reported to facilitate incorporation into multivesicular bodies before secretion [31], whereas the ALIX–Syntenin1–Syndecan1 pathway has been implicated in basolateral HAV release [32]. Despite these advances, whether an nSMase2-dependent ESCRT-independent pathway is associated with extracellular HAV RNA levels has not been determined.

Hepatocytes exhibit a unique functional polarity maintained by tight junctions in which the apical (canalicular) membrane is oriented toward the bile canaliculi and is functionally segregated from the basolateral (sinusoidal) membrane, which faces the bloodstream [33]. Although two independent machineries, the ALIX–Syntenin1–Syndecan1 machinery and the sphingomyelinase-dependent ceramide production machinery, have been reported to contribute to small EV release from the apical and basolateral sides of epithelial cells, respectively [34], the extent of small EV release is dependent on cell type and/or cellular homeostasis [35]. Moreover, the kinetics of HAV apical and basolateral release have been reported to differ among HAV strains [32]. The authors reported that while the HAV HM-175 strain exhibited significantly greater release through the apical membrane than through the basolateral membrane, a fast-replicating HAV HM-175-derived strain harboring a mutation in the VP2 protein, which might facilitate the accessibility of one of the late domains, showed similar levels of release through the apical and basolateral membranes [32]. Further studies are needed to elucidate the differences between HAV strains and release patterns in polarized hepatocytes, as well as their relationships with their replication capacities.

Our findings raise the possibility that host pathways involved in EV biology may contribute to extracellular HAV RNA levels. Such a mechanism may contribute not only to extracellular HAV RNA levels but also to the modulation of the host immune response during infection [36].

The present findings identify the nSMase2-associated pathway as a potential target for future mechanistic studies investigating host-directed antiviral strategies. Although post-exposure prophylaxis with hepatitis A vaccine or immune globulin is effective in preventing HAV infection when administered within 2 weeks of exposure [37], there are currently no approved specific antiviral therapies for established HAV infection, and clinical evidence is insufficient to define the optimal timing of therapeutic intervention. Further studies are needed to determine whether targeting this pathway could have therapeutic potential and whether antiviral intervention during the early phase of infection can prevent progression to severe hepatitis or acute liver failure.

Several limitations of this study should be noted. First, the HAV egress process itself was not directly examined. Second, nSMase2 knockdown was not validated at the protein level. Third, the precise step at which HAV interacts with these pathways relative to the respective drug targets was not directly determined. Fourth, direct EV characterization was not performed; thus, future studies involving EV isolation and characterization are required to confirm whether HAV is directly associated with EVs. Fifth, additional genetic validation, including independent siRNAs, CRISPR-based approaches, or rescue experiments, was not performed. Sixth, apical or basolateral HAV egress was not directly examined. Additionally, further investigations using validated infectivity assays will be necessary to determine whether modulation of this pathway affects the release of infectious HAV particles. Despite these limitations, our findings suggest that an nSMase2-dependent ESCRT-independent pathway may contribute to extracellular HAV RNA levels and, to our knowledge, provide the first evidence supporting this possibility. These findings also suggest that both ESCRT-dependent and ESCRT-independent pathways may contribute to extracellular HAV RNA levels, which may explain why inhibition of a single release pathway results in only partial suppression of extracellular HAV RNA levels.

## 5. Conclusions

The nSMase2-dependent ESCRT-independent pathway may contribute to the presence of extracellular HAV RNA.

## Data Availability

Data are available on request to the corresponding author.

## Abbreviations

The following abbreviations are used in this manuscript:

HAV	hepatitis A virus
ESCRT	endosomal sorting complex required for transport
EVs	extracellular vesicles
STING	stimulator of interferon genes
TSG101	tumor susceptibility gene 101
ALIX	apoptosis-linked gene 2-interacting protein X
CHMP4	charged multivesicular body protein 4
nSMases	neutral sphingomyelinases
Fluc	firefly luciferase
IRES	internal ribosomal entry site
SV40	simian virus 40
RPMI	Roswell Park Memorial Institute medium
FBS	fetal bovine serum
PBS	phosphate-buffered saline
MOI	multiplicity of infection
MTS	dimethylthiazol carboxymethoxyphenyl sulfophenyl tetrazolium
SD	standard deviations
HIV	human immunodeficiency virus
HCV	hepatitis C virus
CSFV	classical swine fever virus
HCMV	Cytomegalovirus
EV71	enterovirus 71
RAS	rat sarcoma virus oncogene
Raf	rapidly accelerated fibrosarcoma
ERK	extracellular signal-regulated kinase
